# Correction to: Weight stigma in the COVID-19 pandemic: a scoping review

**DOI:** 10.1186/s40337-022-00582-1

**Published:** 2022-05-04

**Authors:** Patricia Fortes Cavalcanti de Macêdo, Carina Marcia Magalhães Nepomuceno, Nedja Silva dos Santos, Valterlinda Alves de Oliveira Queiroz, Emile Miranda Pereira, Lucineide da Conceição Leal, Lígia Amparo da Silva Santos, Leonardo Fernandes Nascimento, Poliana Cardoso Martins, Mônica Leila Portela de Santana

**Affiliations:** 1 School of Nutrition, Federal University of Bahia, Salvador, Brazil; 2 Metropolitan Union for the Development of Education and Culture (UNIME), Psychology Course, Salvador, Bahia Brazil; 3 Faculty of Social Sciences, Federal University of Bahia, Salvador, Brazil

## Correction to: Journal of Eating Disorders (2022) 10:44 https://doi.org/10.1186/s40337-022-00563-4

Following publication of the original article [1], the authors identified a typo in the number of original research publications and text and opinion papers included in this review in the following sections:

In the Abstract, the number of original research publications should be “14”. In the Abstract and Results section (second paragraph and Table 1) the number of text and opinion papers should be “15”. In Fig. 2, the number of text and opinion papers published from July to December/2020 should be “7”. In Table 2, the number of “Structural weight stigma” publications should be 25 and the number of “Weight stigma” nomenclature should be 18.

**Table 1:** General characteristics of evidence sources included

Characteristics	No. (%)
**Study type**	
Text and Opinion Papers	15 (42.8)
*Opinion pieces*	4 (11.4)
*Commentary*	3 (8.5)
*Position Statement*	2 (5.7)
*Editorial*	5 (14.2)
*Report*	1 (2.8)
Narrative Reviews	6 (17.1)
Quantitative Studies	6 (17.1)
*Cohort*	3 (8.5)
*Cross-sectional*	3 (8.5)
Qualitative Studies	8 (22.8)
*Interview*	1 (2.8)
*Analysis of media content or public campaign*	6 (17.1)
*Analysis of free-text responses*	1 (2.8)
**Study focus**	35 (100)
Obesity and COVID-19	17 (48.5)
Weight stigma and COVID-19 pandemic	1 (2.8)
Weight stigma in the media during COVID-19 pandemic	2 (5.7)
Eating disorders risk and COVID-19 pandemic	2 (5.7)
Problematization of fatness and COVID-19 pandemic	1 (2.8)
Media/public campaign analysis in the COVID-19 pandemic	6 (17.1)
Weight stigma measured and COVID-19 pandemic	6 (17.1)

**Fig. 2:**
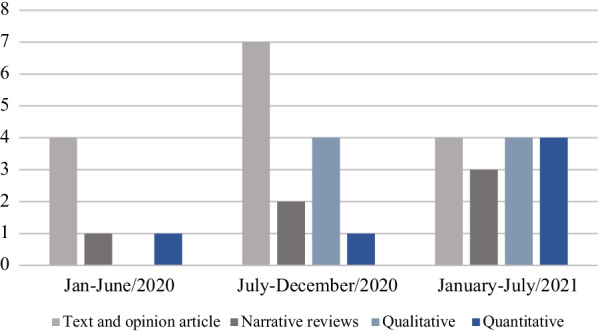
The number of studies published by type of publication throughout the COVID-19 pandemic

**Table 2:** Results mapped according to the three review questions

Type of evidence (No. of studies)	Findings (No. of studies)
**Question 1. Configuration of weight stigma in the pandemic (n = 35)**	
Nomenclature*(35)	Weight stigma (18)
	Obesity stigma (9)
	Weight bias (4)
	Weight discrimination (3)
	Weight bias and stigma (3)
	Others (4)
Cited dimension*(35)	Stigma experienced (pre or during pandemic) (7)
	Self-stigmatization (3)
	Structural weight stigma (25)
	Weight stigmatizing media content (news, social media) (18)
	Weight bias in the healthcare** (13)
	Stigmatizing public health campaigns (5)
	Intersectional stigma (4)
	Not specific / not clear (6)
Measurement* (6)	Experienced/perceived weight stigma (6)
	Single question (3) 3 closed-ended questions (1) 4 closed-ended questions (1) Adapted scale (1)
	Weight stigma internalization (1)
	Scale (1)
	Exposure weight stigma media content (1)
	Single question (1)
Settings/nature* (23)	Media (18)
	Healthcare (13)
	Public campaign (5)
	Public perception of obesity (1)
	Parents and peers (1)
	Online teaching (1)
**Question 2. Consequences/outcomes in the pandemic associated with weight stigma (n = 25)**	
Pre-pandemic/history of weight stigma experiences and pandemic outcomes in quantitative studies* (5)	Decrease in psychological wellbeing indicators in adults (4)
	Greater precautionary behavior to prevent infection in adults (2)
	Association with maladaptive eating in adults (2) ***
	Association with adverse eating behaviours and exercise attitudes in adults (1)
	Predictor of all types of psychological stress in children (1)
Perceived weight stigma during the pandemic and outcomes in quantitative studies (3)	Predictor of all types of psychological stress in children (1)
	Aligned with increased body dissatisfaction during the pandemic in adolescents (1)
Internalized weight stigma measured the pandemic and outcomes in quantitative studies (1)	Association with adverse weight-related health indices in adults (1)
Exposure to weight stigma on social media during the pandemic and outcomes in quantitative studies (1)	Aligned with increased body dissatisfaction during the pandemic in adolescents (1)
Weight stigma and associated consequences in qualitative studies (1)	Decreased ability to engage in obesity treatment during the pandemic because of self-stigmatization in adults (1)
Weight stigma and associated consequences in reviews and text and opinion documents* (18)	Impediment/delay in accessing information/health services in the pandemic (10) as a consequence of the weight stigma suffered in the healthcare (5), previous experiences of weight stigma (3), high stigma/perceived vulnerability in the pandemic (1), concern to be included as a burden to health system(1) and intersection of vulnerabilities in the pandemic (1)
	Worst outcomes for COVID-19 (8) associated with reluctance/delay in seeking health services in the pandemic (5), poorer quality of care for PwO in the pandemic (1), and intersections of vulnerabilities in the pandemic (3)
	The worsening quality of care provided in the pandemic (2) associated with weight bias in health professionals during the pandemic (2) and lack of health equipment/accommodations for PwO (1)
	Risk of illness by COVID-19 associated with rationing of COVID-19 care or resources for fat people (1)
	Deepening health inequities in face of weight stigma (1)
	The relegation of bariatric surgery as an indication of systematic bias and discrimination towards PwO (1)
	Increase ssusceptibility to disordered or maladaptive eating (6) associated with exposure to stigmatizing weight content on social media in the pandemic (2), stigmatization of obesity in public campaigns (1) , PwO stigmatization history (1); children’s bullying in online education (1), previous experiences of weight stigma (1)
**Question 3. Perceptions/experiences of weight stigma in PwO in the pandemic (n = 2)**	
Perceptions/experiences in a qualitative study(1)	Difficulty in getting involved in the treatment of obesity in the pandemic associated with weight self-stigma (1)
Perceptions/experiences in a text and opinion document* (1)	Feeling like are low priority than any other condition as a result of stigma on social media during the pandemic (1)
	Impediment to exercise/buy food in the pandemic associated with stigma or shame (1)

*PwO* people with obesity

*More than one finding may have been identified in a single document

**Evidence of lack of structure or equipment for people with obesity, reluctance to seek help, or weight bias by health professionals

***Engaging in more eating to cope or binge eating

The correct Tables 1, 2 and Fig. 2 have been included in this correction, and the original article has been updated.
